# Mechanical and Pharmacological Revascularization Strategies for Prevention of Microvascular Dysfunction in ST-Segment Elevation Myocardial Infarction: Analysis from Index of Microcirculatory Resistance Registry Data

**DOI:** 10.1155/2020/5036396

**Published:** 2020-07-09

**Authors:** Ji-Hun Jang, Man-Jong Lee, Kyu-Yong Ko, Jin-Hee Park, Yong-Soo Baek, Kwon Sung-Woo, Sung-Hee Shin, Seong-Ill Woo, Dae-Hyeok Kim, Young Ju Suh, Jun Kwan, Sang-Don Park

**Affiliations:** ^1^Division of Cardiology, Department of Internal Medicine, Inha University Hospital, Incheon, Republic of Korea; ^2^Department of Biomedical Sciences, Inha University Hospital, Inha University School of Medicine, Incheon, Republic of Korea

## Abstract

**Objectives:**

We aimed to identify mechanical and pharmacological revascularization strategies correlated with the index of microcirculatory resistance (IMR) in ST-elevation myocardial infarction (STEMI) patients.

**Background:**

Microvascular dysfunction (MVD) after STEMI is correlated with infarct size and poor long-term prognosis, and the IMR is a useful analytical method for the quantitative assessment of MVD. However, therapeutic strategies that can reliably reduce MVD remain uncertain.

**Methods:**

Patients with STEMI who underwent primary percutaneous coronary intervention (PCI) were enrolled. The IMR was measured with a pressure sensor/thermistor-tipped guidewire immediately after primary PCI. High IMR was defined as values ≥66^th^ percentile of IMR in enrolled patients (IMR > 30.9 IU).

**Results:**

A total of 160 STEMI patients were analyzed (high IMR = 54 patients). Clinical factors for Killip class (*P*=0.006), delayed hospitalization from symptom onset (*P*=0.004), peak troponin-I level (*P*=0.042), and multivessel disease (*P*=0.003) were associated with high IMR. Achieving final thrombolysis in myocardial infarction myocardial perfusion grade 3 tended to be associated with low IMR (*P*=0.119), whereas the presence of distal embolization was significantly associated with high IMR (*P*=0.034). In terms of therapeutic strategies that involved adjusting clinical and angiographic factors associated with IMR, preloading of third-generation P2Y12 inhibitors correlated with reducing IMR value (*β* = −10.30, *P* < 0.001). Mechanical therapeutic strategies including stent diameter/length, preballoon dilatation, direct stenting, and thrombectomy were not associated with low IMR value (all *P* > 0.05), and postballoon dilatation was associated with high IMR (*β* = 8.30, *P*=0.020).

**Conclusions:**

In our study, mechanical strategies were suboptimal in achieving myocardial salvage. Preloading of third-generation P2Y12 inhibitors revealed decreased IMR value, indicative of MVD prevention.

## 1. Introduction

ST-elevation myocardial infarction (STEMI) is usually caused by complete occlusion of a major epicardial coronary artery and results in myocardial ischemia and cell death. Early reopening of the culprit artery by primary percutaneous coronary intervention (PCI) is now considered as the cornerstone of the treatment [1–3]. However, although patients with STEMI restored normal coronary flow after primary PCI in many cases, they failed to achieve myocardial microvascular reperfusion, resulting in poor clinical outcome [4, 5]. Many previous studies demonstrated that coronary microvascular dysfunction (MVD) was correlated with infarct size, and the presence of MVD was associated with an increased risk of cardiovascular events. Distal embolization of atheromatous debris, swelling of cardiomyocytes associated with interstitial edema, and reperfusion-related myocardial injury after primary PCI is considered as the major mechanism of microvascular damage [5–10].

The index of microcirculatory resistance (IMR) can provide a quantitative assessment of the microvascular function of epicardial stenosis and hemodynamic condition independently. IMR is considered an independent, powerful predictor of microvascular damage in STEMI [11–15]. Recent studies demonstrated that IMR assessed immediately after primary PCI is well correlated with the recovery of left ventricular function in STEMI [1, 7].

Through imaging tools or invasive coronary physiological indices, many studies have attempted to prove the therapeutic strategies of mechanical and pharmacological revascularization that can reduce microvascular damage from myocardial infarction. However, there are no definitive treatments for myocardial infarction that can reliably reduce microvascular damage, and some current therapeutic strategies remain controversial [4, 16–18].

Therefore, we aimed to identify mechanical and pharmacological revascularization strategies that prevent MVD in patients with STEMI using IMR that can assess microvascular integrity with primary PCI.

## 2. Materials and Methods

### 2.1. Study Population

We retrospectively reviewed consecutive patients with STEMI who underwent primary PCI with coronary physiological measurements between May 2009 and June 2016 at the clinic of a tertiary referral center (Inha University Hospital, South Korea).

STEMI was diagnosed in patients with symptoms that presented as myocardial ischemia with ST-segment elevation on electrocardiography (ECG) and subsequent release of cardiac biomarkers. ST-elevation was defined as new ST elevation at *J* point in two contiguous leads of ≥2 mm (0.2 mV) in men or ≥ 1.5 mm (0.15 mV) in women in leads V2∼3 and/or of ≥1 mm (0.1 mV) in other contiguous chest leads of the limb leads [2, 3]. We enrolled the patients who had developed STEMI within 12 hours (symptom to hospital time < 12 hours) and had been successfully treated by primary PCI of the infarct-related artery with a modern drug-eluting stent. The patients who had unprotected left main coronary artery disease or the culprit lesion at a side branch, stent thrombosis, high-degree atrioventricular block, cardiogenic shock with Killip class IV, contraindication to adenosine, previous cerebrovascular accident or myocardial infarction, and final thrombolysis in myocardial infarction (TIMI) grade 0/1 were excluded.

This study design was approved by the Institutional Review Board of Inha University Hospital, Incheon, South Korea, and was conducted in compliance with the ethical principles outlined in the Declaration of Helsinki (INHAUH2020-04-057). The written informed consent was obtained from all patients.

### 2.2. Echocardiographic Analysis and Cardiac Biomarkers

Transthoracic echocardiography was performed using commercially available instruments less than 24 hours after primary PCI. Echocardiographic parameters were measured following the American Society of Echocardiography guidelines [19]. Left ventricular ejection fraction (LVEF) was calculated by the biplane Simpson method. Regional wall motion abnormality was obtained according to the recommendations of current guidelines. An experienced cardiologist who had no information of IMR accessed the wall motion score index (WMSI). WMSI was assessed in a 16-segment model and calculated as the sum of all scores divided by the number of segments visualized. LV mass and LV mass indexed to body surface area were estimated by LV cavity dimension and wall thickness at end-diastole.

Baseline *N*-terminal pro-*B*-type natriuretic peptide (NT-proBNP) level was checked, and cardiac enzymes were measured before and after PCI and the highest value during follow-up was assessed.

### 2.3. Angiographic Analysis and Treatment Strategies for ST-Elevation Myocardial Infarction

All patients received preloading of dual-anticoagulation drugs (300 mg of aspirin and 600 mg of clopidogrel or 180 mg of ticagrelor or 60 mg of prasugrel) before primary PCI. All patients were administered intravenous continuous infusion (100 IU/Kg) following a bolus injection of unfractionated heparin (5,000 IU). Intravenous morphine was used depending on the clinician's decision before or during PCI. After a loading dose of anticoagulation, primary PCI was performed in accordance with the current guideline [3]. A full range of commercially available guiding catheters, balloon catheters, and guide wires were used. Pre/postballoon dilation, direct stenting, or thrombus aspiration was performed based on the physician's discretion. Thrombus aspiration was performed from >2 passages across the lesion using an Export Advance aspiration catheter (Medtronic, Inc., Minneapolis, MN, USA) before stent insertion. Glycoprotein (GP) IIb/IIIa inhibitors were administered as bailout therapy in the events of slow- or no-reflow phenomenon after revascularization. Pre- and post-TIMI grade and TIMI myocardial perfusion grade (TMPG) were assessed using grades 0–3 based on final cine images obtained after a successful primary PCI. TIMI thrombus grade, collateral flow grade, and presence of distal embolization were obtained from the angiographic findings [20, 21]. Distal embolization was defined as a distal filling defect with a sudden “cut-off” in one of the coronary branches of the culprit artery, distal to the angioplasty site [9].

Stent size/diameter and periprocedural techniques including pre/postballoon dilatation, direct stenting, and thrombus aspiration were defined as “mechanical strategies.” “Pharmacologic strategies” included loading of dual antiplatelet, use of GP IIb/IIIa inhibitors, and morphine use.

### 2.4. Physiological Assessment: IMR Study

**Figure 1:**
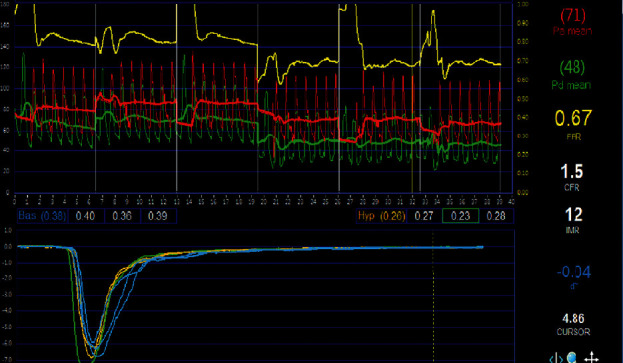
Thermodilution curves under resting conditions (yellow lines) and during hyperemia (blue lines) induced by intravenous adenosine infusion. A graphical representation of the baseline and hyperemia (seconds) thermodilution curves is seen in the catheterization lab at the time of percutaneous coronary intervention that was displayed on the RADI analyzer (Radi Medical Systems, Uppsala, Sweden).

IMR was measured with a pressure sensor/thermistor-tipped guide wire (Radi Pressure Wire 5; Radi Medical Systems, Uppsala, Sweden) at the culprit lesion immediately after primary PCI (Figure 1). The pressure sensor/thermistor-tipped guide wire was initially calibrated outside the body and positioned in the distal two-thirds of the culprit vessel after equalizing to the guiding catheter [12, 22]. Intracoronary nitroglycerine (200 *μ*g) was administered and hyperemia was induced by administration of adenosine infusion (140 *μ*g/kg∙min) via femoral or antecubital vein. After achieving maximal hyperemia, the mean hyperemic transit time was recorded by averaging the value after rapid injection of 3 mL of room-temperature saline through the coronary catheter. At the same time, mean aortic pressure (Pa) and distal coronary arterial pressure (Pd) were obtained. Fractional flow reserve (FFR) was calculated by using the following equation:

(1) $$\begin{array}{c} \text{FFR}=\frac{\mathrm{distal}\mathrm{coronary}\mathrm{pressure}}{\mathrm{aortic}\mathrm{pressure}}. \end{array}$$

The IMR was calculated as using the following equation:

(2) $$\begin{array}{c} \mathrm{IMR=distal}\mathrm{pressure\times Tmn}\left(\mathrm{mean}\mathrm{transit}\mathrm{time}\right)\mathrm{during}\mathrm{hyperemia}. \end{array}$$

Additionally, thermodilution coronary flow reserve (CFR) was calculated using the following equation:

(3) $$\begin{array}{c} \text{CFR}=\frac{\mathrm{hyperemic}\mathrm{Tmn}}{\mathrm{resting}\mathrm{Tmn}}. \end{array}$$

Vessels with severe stenosis (distal coronary pressure ≤ 60 mmHg) and collateral flow were excluded from the analysis [22]. All IMR values were corrected by using Yong's formula (corrected IMR (IMRcorr) = Pa x Tmn x ([1.35 x Pd/Pa]−0.32). We defined “High IMR” as values ≥ 66th percentile of IMRcorr in the enrolled patients. In our study, “High IMR” was IMRcorr ≥ 30.9 U.

### 2.5. Clinical Outcome

We assessed 3-year major advanced cardiac events (MACE) for the study population. MACE was defined as the composite of cardiovascular (CV) death, hospitalization because of heart failure (HF), target lesion revascularization (TLR), stent thrombosis, nonfatal myocardial infarction (MI), stroke, and major bleeding.

### 2.6. Statistical Analyses

Data were expressed for continuous variables as mean ± standard deviation (SD) or median with interquartile range (IQR), as needed. Categorical variables were expressed as counts and percentages. Student's *t*-test and Pearson's Chi-square test were used to compare each parameter as needed. The Mann–Whitney *U* test was used for skewed variables, and Fisher's exact test was used when the expected frequency was lower than 5. To determine the variables related to IMR value, linear regression analysis was performed. The final multiple regression model was made by stepwise forward regression based on a *Pvalue* of 0.2 and the clinical significance. The therapeutic strategies associated with IMR value were analyzed using multiple linear regression analysis. In addition, therapeutic strategies that were significantly associated with IMR value were adjusted for parameters that considered significant relation with IMR value on the previous multivariate model.

For all tests, a *Pvalue* less than 0.05 was considered statistically significant. All statistical analyses were performed with *R* statistical software (version 3.4.1; *R* Foundation for Statistical Computing, Vienna, Austria).

## 3. Results

### 3.1. Baseline Characteristics

**Figure 2:**
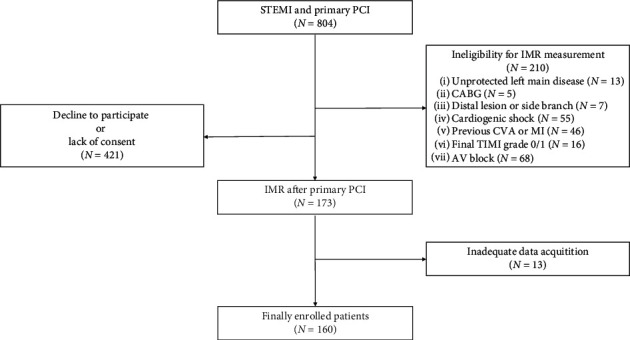
Study flowchart.

Of the 804 patients with STEMI, 210 patients could not perform coronary physiological measurements according to the exclusion criteria. Additionally, 421 patients did not agree with the IMR study or were unable to provide informed consent. Finally, 160 patients were enrolled in our study (Figure 2).

The baseline clinical data are as follows (Table 1). The overall mean age was 56 ± 11 years, 141 patients (88.1%) were men, and 6 patients (3.8%) showed Killip class III upon admission.

The High IMR group was older than the Low IMR group (60 ± 11 years versus 54 ± 11 years, *P*=0.001). Medical history was comparable between the two groups. While there was no difference for the door to balloon time, the High IMR group showed delayed hospitalization after symptom development (144.0 [71.0–360.0]) min versus 95.0 [(60.0–174.0] min, *P*=0.003 for symptom onset to hospital time) and delayed revascularization (235.0 [159.0–397.0] min versus 162.0 [115.0–265.0] min, *P*=0.001 for symptom onset to balloon time). The High IMR group showed a greater decrease in LV systolic function (LVEF, 44.7 ± 6.8% versus 47.3 ± 7.0%, *P*=0.028) and elevated E/e' (10.5 [9.3–12.5] versus 9.8 [8.0–11.2], *P*=0.024). WMSI was more elevated in the High IMR group (1.7 [1.3–1.8] versus 1.5 [1.2–1.8], *P*=0.040). The peak levels of creatine kinase (CK) and creatine kinase muscle/brain (CK-MB) were more elevated in the High IMR group (peak CK, 3102.0 [1406.0–5580.0] IU/L versus 2020.0 [729.0–3897.0] IU/L, *P*=0.028; peak CK-MB, 289.4 [129.0–465.9] ng/mL versus 188.0 [73.0–334.0] ng/mL, *P*=0.010), whereas troponin-I level was comparable (*P*=0.084). NT-proBNP level was higher in the High IMR group but statistically nonsignificant.

### 3.2. Coronary Angiography and Physiologic Studies

The results of coronary angiography and physiological studies are displayed in Table 2. The multivessel disease was more frequent in the High IMR group (53.7% vs. 30.2%, *P*=0.006). However, there was no significant difference in the vessel territory of the culprit lesion between the two groups. A hundred patients (62.5%) showed initial TIMI grade 0/1 flow before PCI, 144 patients (90.0%) achieved final TIMI grade 3 flow, and 112 patients (70.0%) showed final TMPG 3. Twenty-six (16.2%) patients showed distal embolization and High IMR group was observed more often (31.5% versus 8.5%, *P* < 0.001). Fifteen patients (9.4%) showed no-reflow phenomenon during PCI. The High IMR group could not achieve final TIMI 3 flow and final TMPG 3 compared with the Low IMR group (77.8% versus 96.2%, *P*=0.001 and 46.3% versus 82.1%, *P* < 0.001, respectively). Although TIMI thrombus grade and collateral flow grade were comparable between the two groups, the presence of distal embolization was greater in the High IMR group (14.8% versus 6.6%, *P* < 0.001).

In physiological assessment, there were significant differences in resting Tmn and hyperemic Tmn (0.7 [0.6–1.0] versus 0.4 [0.3–0.6], *P* < 0.001; 0.6 [0.4–0.8] versus 0.2 [0.2–0.3], *P* < 0.001, respectively). The overall value of FFR was similar between the two groups, whereas CFR was lower in the High IMR group (1.3 [1.0–1.8] versus 1.8 [1.2–2.6], *P* < 0.001). The overall median IMR was 22.4 [13.9–35.0] U.

### 3.3. Clinical Outcomes of the Study Population

As 7 patients were lost during follow-up, we analyzed 153 patients for 3-year MACE. Three-year MACE occurred in 17 (11.1%) patients and were comparable between the two groups (15.4% versus 8.9%, *P*=0.279). CV death occurred in 3 (2.0%) patients who were included only in the High IMR group (*P*=0.038). Other events including hospitalization for HF, TLR, stent thrombosis, nonfatal MI, stroke, and major bleeding were comparable (Table 3).

### 3.4. Therapeutic Strategies for STEMI

Mechanical and pharmacological treatment strategies were accessed in the study population (Table 4). All patients were implanted with third-generation drug-eluting stents. The stent size and length were comparable between the two groups. There was no significant difference in the treatment of balloon angioplasty (68.5% versus 76.4%, *P*=0.377 for preballoon dilatation; 16.7% versus 10.4%, *P*=0.376 for postballoon dilatation). Forty-two patients (26.2%) underwent direct stenting and 75 patients (46.9%) underwent aspiration thrombectomy. Thirty-one patients (19.4%) underwent a combination of aspiration thrombectomy with GP IIb/IIIa inhibitors. All mechanical therapeutic strategies showed no significant difference between the two groups.

All patients received preloading with 300 mg of aspirin. The High IMR group showed a higher incidence of clopidogrel use than third-generation P2Y12 inhibitors (79.6% versus 60.4%, *P*=0.023). The GP IIb/IIIa inhibitor was more frequently used in the High IMR group than in the Low IMR group (27.8% versus 17.9%, *P*=0.216). In terms of morphine use, both groups showed almost equal proportions (51.9% versus 64.2%, *P*=0.183).

### 3.5. Predictors of Microvascular Dysfunction and Therapeutic Strategies Preventing Microvascular Dysfunction

As a result of analyzing the clinical, echocardiographic, and angiographic parameters, higher Killip class, delayed symptom-onset-to-hospital time, higher peak troponin-I level, presence of multivessel disease, and occurrence of distal embolization were correlated with increasing IMR value (Table 5).

We analyzed each therapeutic strategy for the association of IMR value using linear regression analysis (Table 6). In mechanical strategies, increasing stent diameter was not significantly associated with low IMR value (*β* = −8.00, *P*=0.057). Longer stent length and postballoon dilatation were associated with higher IMR value (*β* = 0.31, *P*=0.046 and *β* = 10.44, *P*=0.015, respectively). Preballoon dilatation, direct stenting, and thrombectomy showed no significant association with IMR value. In pharmacological strategies, the loading of third-generation P2Y12 inhibitors was significantly associated with low IMR value (*β* = −7.24, *P*=0.017). The use of morphine and GP IIb/IIIa inhibitor was not associated with low IMR value.

In multiple regression analysis for therapeutic strategies using stepwise regression, stent diameter and loading of third-generation P2Y12 inhibitors were negatively correlated with IMR value (*β* = −9.39, *P*=0.018 and *β* = −10.69, *P* < 0.001, respectively). Postballoon dilatation and the use of GP IIb/IIIa inhibitor were positively correlated with IMR value (*β* = 11.40, *P*=0.008 and *β* = 8.51, *P*=0.013, respectively).

**Figure 3:**
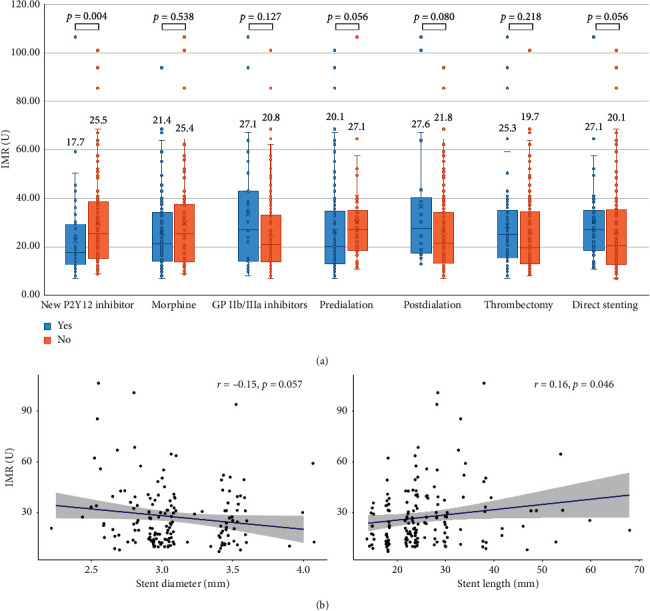
Comparison of the index of microcirculatory resistance according to therapeutic strategies. (a) Each therapeutic strategy was displayed as box plots. The displayed number means the median value. (b) The association between IMR value and stent diameter/length was displayed as scatter plot.

We analyzed each therapeutic strategy that was significantly associated with IMR value, adjusting for age, sex, and parameters for the previous multiple analysis model in Table 5. Of the stent diameter/length, postballoon dilatation, third-generation P2Y12 inhibitors, and use of GP IIb/IIa inhibitors, only loading of third-generation P2Y12 inhibitors were in significant association with low IMR value (*β* = −10.28, *P* < 0.013). A comparison of IMR according to therapeutic strategies is shown in Figure 3.

## 4. Discussion

The main objective of this study was to determine the therapeutic strategy to reduce MVD in patients with STEMI. The major findings of this study were as follows. (1) Higher Killip class, higher peak troponin-I level, presence of multivessel disease, distal embolization, and delayed reperfusion time were associated with higher IMR value. This finding was consistent with those in previous studies. (2) The mechanical therapeutic strategy had no significant association with lower IMR value. (3) In pharmacological strategies, the preloading of third-generation P2Y12 inhibitors showed a significant association with lower IMR value.

### 4.1. Clinical Parameters Increasing the Risk of MVD

It has been reported that the burden of ischemic myocardia is a crucial factor in determining the occurrence of MVD [5–9]. The extent of myocardial injury is associated with an increased mortality risk.

Many previous articles show that the IMR provides coronary microcirculation and is independently predictive of LV function and infarct volume and microvascular damage after STEMI [6, 11–14, 23, 24]. Although the exact cut-off value of IMR representing the microvascular damage varies, the key point is that higher IMR value is associated with MVD and ischemic burden of the myocardium. Therefore, high IMR value can represent the MVD of the myocardium.

Previous studies have reported that the occurrence of heart failure (higher Killip class), multivessel disease, and a longer duration from symptom onset was associated with poor prognosis [15, 25]. Also, the IMR value well correlates with peak troponin-I concentration [24]. Our result is consistent with previous findings, indicating that these clinical factors correlate well with higher IMR values.

TMPG reflected the degree of impaired myocardial perfusion. Impaired TMPG has been associated with a greater coronary thrombus burden, larger infarct size, and poorer salvage indices [5, 26]. Achieving the final TMPG, grade 3 was associated with lower IMR value in our study, whereas initial and final TIMI grade showed no significant findings on multivariate analysis. Our findings also support that TMPG, which assesses microvascular clearance, was more sensitive to microvascular function than TIMI flow, which reflected epicardial coronary flow. Distal embolization is related to reduced myocardial reperfusion, more extensive myocardial damage, and a poor prognosis [9]. Like previous study results, our data showed that the presence of distal embolization was significantly correlated with higher IMR value. This finding suggests that an additional therapeutic strategy is necessary to prevent or treat distal embolization during PCI.

### 4.2. Which Therapeutic Approaches Can Reduce MVD?

Reducing periprocedural myocardial injury and enhancing myocardial salvage during primary PCI in patients with STEMI have been a major concern for treatment strategies. Recent studies have shown that distal embolization occurs in 11% of patients STEMI treated by conventional primary PCI and that occurrence increases the risk of heart failure [27]. Therefore, various mechanical strategies have been developed over the years to reduce distal embolization during primary PCI. However, although most mechanical strategies are still being investigated for potential clinical benefit, their clinical efficacy remains unproven, and their use in primary PCI routine operations is quite limited [28]. The previous study showed that direct stenting provides better immediate TIMI flow and is a safe and feasible method for selected lesions compared with conventional stenting [29]. However, lesions eligible for direct stenting were shorter and were less complicated than those requiring predilatation. Also, there was a limitation that it was difficult to measure the length of the stent or predict the lesion of stenosis [28]. Direct stenting showed no preventive effect of lowering IMR value in our study. The effect of direct stenting was quite limited and may be eligible only in less complicated culprit lesions.

Various studies have been conducted on the effectiveness of aspiration thrombectomy. However, these promising results did not lead to clinical benefit in the previous randomized trials [17, 30]. Thus, in the light of current research, the routine use of aspiration thrombectomy is not recommended in recent guidelines [2]. We attempted aspiration thrombectomy alone or combination therapy with GP IIb/IIIa inhibitors. However, these procedural techniques showed no association of lower IMR value.

Although recent meta-analysis showed that intracoronary GP IIb/IIIa inhibitors might be reasonable as bailout therapy in high-risk patients with STEMI, there is no evidence that routine use during primary PCI could reduce myocardial ischemic size [31]. The use of GP IIb/IIIa inhibitors showed no association with IMR value in our study. We speculate that concomitant use with thrombus aspiration or use in patients with no reflow may have reduced the effect of GP IIb/IIIa.

A previous cohort study showed that intravenous morphine infusion had a positive effect on preventing myocardial reperfusion injury. They suggested that morphine inhibits the opening of mitochondrial permeability transition pores (mPTP) mediated by the activation of delta-opioid receptors. Opioid receptor activation-triggered postconditioning may protect the myocardial injury by targeting mPTP [32]. However, another randomized controlled trial demonstrated that morphine did not show the preventive effect of myocardial salvage. They suggested that morphine use is associated with a slower uptake, delayed onset of action, and diminished effects of oral antiplatelet agents [33]. We demonstrated that intravenous morphine use during primary PCI did not differ between the two groups. Our result suggests that morphine did not reduce myocardial infarct burden. However, as there was no clear research on the relationship between morphine use and MVD in patients with STEMI, clarifying their relationship must be investigated by future studies.

Third-generation P2Y12 inhibitors, prasugrel and ticagrelor, are recommended based on improved clinical outcomes and more potent platelet inhibition compared with clopidogrel in acute coronary syndrome (ACS) [34]. When compared with potent inhibition of platelet function, ticagrelor and prasugrel inhibit approximately 94% and 90%, respectively, in those with aspirin, whereas clopidogrel typically achieves a maximum of only 50% platelet inhibition in combination with aspirin in ACS. Ticagrelor and prasugrel are faster than clopidogrel in reaching its peak concentration (peak effect after loading dose at 2 hours for ticagrelor, 4 hours for prasugrel, and 6 hours for clopidogrel) and has been shown to increase adenosine plasma concentration associated with an inhibition of adenosine uptake of red blood cells, stimulating vasodilatation [35–37]. In our study, microvascular injury was reduced more in the third-generation P2Y12 inhibitors group. Our results support that third-generation P2Y12 inhibitor is more effective in lowering IMR value than clopidogrel in patients with STEMI and this finding represents the protective effect of MVD.

## 5. Study Limitation

Our data did not show any beneficial effect of mechanical strategies on lowering IMR value. Although the larger stent diameter and shorter stent size tend to be associated with lower IMR values in multiple regression analyses which suggested that securing the maximal stent lumen and minimizing the stent length are important for myocardial salvage, our findings suggested that conventional mechanical procedures did not reduce distal embolization effectively. However, as the interventional approach is driven by the operator's decision depending on the nature of the culprit lesion, our mechanical therapeutic strategy cannot be generalized. Thus, selection bias dependent on the interventionist may be reflected in the establishment of treatment strategies, and these variables may play a role as confounders. Moreover, because of data from a relatively small number of patients and a single referral tertiary institute were used, our study participants may be a skewed and selected population based on disease severity and comorbidities, rather than representing the general population. In our study, IMR alone was used to evaluate MVD and no other image tools were used to measure the magnitude of infarct size. Although several studies have shown that IMR is an independent predictor of MVD, radionuclide myocardial perfusion imaging 99mTechnetium Sestamibi single-photon emission tomography and cardiovascular magnetic resonance are still gold standards for measuring myocardial salvage [23]. Further studies would need to be done to evaluate whether the relationship between our therapeutic strategies and MVD hold true in a larger study population, over a long-term follow-up period.

## 6. Conclusions

In our study, mechanical strategies were suboptimal in achieving myocardial salvage. Only preloading of third-generation P2Y12 inhibitors was associated with low IMR value which represents a trend of MVD prevention in STEMI patients. Therefore, it is necessary to use third-generation P2Y12 inhibitors according to the current guidelines, and novel procedural techniques should be developed to reduce MVD in patients with STEMI.

## Figures and Tables

**Table 1:** Clinical characteristics of the study population.

Parameter	Total (*N* = 160)	High IMR (*N* = 54)	Low IMR (*N* = 106)	*P* value
Clinical characteristics				
Age (years)	56 ± 11	60 ± 11	54 ± 11	0.001
Male, *n* (%)	141 (88.1%)	45 (83.3%)	96 (90.6%)	0.281
Current smoking, *n* (%)	115 (71.9%)	36 (66.7%)	79 (74.5%)	0.353
BMI	24.6 [22.9–26.6]	25.0 [21.9–26.9]	24.6 [23.4–26.5]	0.734
SBP (mmHg)	134.9 ± 23.8	137.7 ± 25.3	133.5 ± 23.1	0.299
DBP (mmHg)	84.4 ± 16.2	85.3 ± 17.2	84.0 ± 15.7	0.637
HR (beats/min)	76.8 ± 15.7	73.7 ± 15.6	78.4 ± 15.6	0.075
Killip class				0.053
I	122 (76.2%)	35 (64.8%)	87 (82.1%)	
II	32 (20.0%)	16 (29.6%)	16 (15.1%)	
III	6 (3.8%)	3 (5.6%)	3 (2.8%)	

Medical history, *n* (%)				
Hypertension	77 (48.1%)	31 (57.4%)	46 (43.4%)	0.131
Diabetes mellitus	49 (30.6%)	20 (37.0%)	29 (27.4%)	0.283
Dyslipidemia	68 (42.5%)	21 (38.9%)	47 (44.3%)	0.624

Time to reperfusion, min				
DTB, min	68.0 [57.0–80.0]	69.5 [52.0–83.0]	68.0 [59.0–80.0]	0.779
SHT, min	120.0 [60.0–210.0]	144.0 [71.0–360.0]	95.0 [60.0–174.0]	0.003
SBT, min	188.5 [125.5–293.0]	235.0 [159.0–397.0]	162.0 [115.0–265.0]	0.001

Echocardiographic analysis				
LVEDD (mm)	49.0 [46.0–51.0]	48.0 [45.0–51.0]	49.0 [46.0–51.0]	0.664
LVESD (mm)	36.0 [33.0–38.0]	34.0 [32.0–39.0]	36.0 [33.0–38.0]	0.489
LVEF (%)	46.5 ± 7.0	44.7 ± 6.8	47.3 ± 7.0	0.028
E/e'	10.1 [8.4–11.9]	10.5 [9.3–12.5]	9.8 [8.0–11.2]	0.024
WMSI	1.6 [1.2–1.8]	1.7 [1.3–1.8]	1.5 [1.2–1.8]	0.040
LV mass (g)	188.1 [158.2–213.6]	193.5 [147.8; 220.6]	188.1 [158.8; 207.3]	0.690
LV mass index (g/m^2^)	104.8 [88.9; 116.7]	107.9 [89.2; 119.3]	103.4 [88.9; 114.5]	0.424

Cardiac biomarkers				
CK peak, IU/L	2373.0 [774.0–4276.0]	3102.0 [1406.0–5580.0]	2020.0 [729.0–3897.0]	0.028
CK-MB peak, ng/mL	224.9 [78.2–370.0]	289.4 [129.0–465.9]	188.0 [73.0–334.0]	0.010
Troponin-I peak, ng/mL	82.7 [21.0–148.4]	100.0 [47.0–250.0]	66.0 [17.4–122.5]	0.084
NT-proBNP, pg/mL	70.0 [23.0–255.5]	106.0 [36.0–293.5]	51.5 [21.0–186.0]	0.054

Renal function				
BUN, mg/dL	15.3 [12.9–18.3]	14.9 [12.2–18.5]	15.4 [13.3–18.1]	0.582
Creatinine, mg/dL	1.0 [0.9–1.2]	1.0 [0.9–1.2]	1.0 [0.9–1.1]	0.543
eGFR (ml/min/1.73 m^2^)	77.5 [68.0–91.1]	72.0 [62.7–91.2]	79.5 [71.3–91.1]	0.081
eGFR < 60 ml/min/1.73 m^2^, *n* (%)	18 (11.2%)	9 (16.7%)	9 (8.5%)	0.184

BMI, body mass index; BUN, blood urea nitrogen; CK, creatine kinase; CK-MB, creatine kinase muscle/brain; DBP, diastolic blood pressure; DTB, door to balloon time; eGFR, estimated glomerular filtration rate; HR, heart rate; IMR, index of microcirculatory resistance; LV, left ventricle; LVEDD, left ventricular end-diastolic diameter; LVEF, left ventricular ejection fraction; LVESD, left ventricular end-systolic diameter; NT-proBNP, N-terminal prohormone of brain natriuretic peptide; SBP, systolic blood pressure; SBT, symptom onset to balloon time; SHT, symptom onset to hospital time; WMSI, wall motion score index.

**Table 2:** Coronary angiography findings and physiological assessment of study population.

Parameter	Total (*N* = 160)	High IMR (*N* = 54)	Low IMR (*N* = 106)	*P* value
Angiographic parameters				
Multivessel, *n* (%)	61 (38.1%)	29 (53.7%)	32 (30.2%)	0.006

Vascular territory, *n* (%)				
LAD	114 (71.2%)	41 (75.9%)	73 (68.9%)	0.454
LCX	14 (8.8%)	6 (11.1%)	8 (7.5%)	0.647
RCA	32 (20.0%)	7 (13.0%)	25 (23.6%)	0.168

Initial TIMI grade, *n* (%)				
TIMI 0–1	100 (62.5%)	39 (72.2%)	61 (57.5%)	0.101
TIMI 2	43 (26.9%)	13 (24.1%)	30 (28.3%)	0.703
TIMI 3	17 (10.6%)	2 (3.7%)	15 (14.2%)	0.079

Final TIMI grade, *n* (%)				
TIMI 2	16 (10.0%)	12 (22.2%)	4 (3.8%)	0.001
TIMI 3	144 (90.0%)	42 (77.8%)	102 (96.2%)	0.001

Final TMPG, *n* (%)				
TMPG 2	45 (28.1%)	27 (50.0%)	18 (17.0%)	<0.001
TMPG 3	112 (70.0%)	25 (46.3%)	87 (82.1%)	<0.001

TIMI thrombus grade, *n* (%)				0.215
Grade 1	4 (2.5%)	2 (3.7%)	2 (1.9%)	
Grade 2	12 (7.5%)	3 (5.6%)	9 (8.5%)	
Grade 3	25 (15.6%)	4 (7.4%)	21 (19.8%)	
Grade 4	36 (22.5%)	13 (24.1%)	23 (21.7%)	
Grade 5	83 (51.9%)	32 (59.3%)	51 (48.1%)	

Collateral flow grade, *n* (%)				0.215
Rentrop 1	115 (71.9%)	41 (75.9%)	74 (69.8%)	
Rentrop 2	35 (21.9%)	12 (22.2%)	23 (21.7%)	
Rentrop 3	1 (0.6%)	0 (0.0%)	1 (0.9%)	
Distal embolization, *n* (%)	26 (16.2%)	17 (31.5%)	9 (8.5%)	<0.001
No reflow, *n* (%)	15 (9.4%)	8 (14.8%)	7 (6.6%)	0.162

Physiologic parameters				
Pa (hyp), mm Hg	86.0 ± 16.3	84.9 ± 17.4	86.5 ± 15.7	0.564
Pd (hyp), mm Hg	78.5 ± 15.1	78.7 ± 15.7	78.5 ± 14.9	0.915
Tmn at rest, sec	0.5 [0.3–0.8]	0.7 [0.6–1.0]	0.4 [0.3–0.6]	<0.001
Tmn, hyperemia, sec	0.3 [0.2–0.4]	0.6 [0.4–0.8]	0.2 [0.2–0.3]	<0.001
FFR	0.9 [0.9–1.0]	0.9 [0.9–1.0]	0.9 [0.9–1.0]	0.256
CFR	1.6 [1.1–2.4]	1.3 [1.0–1.8]	1.8 [1.2–2.6]	<0.001
IMR, U	22.1 [13.7–34.9]	39.8 [34.9–52.2]	16.5 [12.5–22.1]	<0.001
IMRcorr, U	22.4 [13.9–35.0]	40.2 [34.9–52.2]	16.4 [12.4–22.2]	<0.001

CFR, coronary flow reserve; FFR, fractional flow reserve; Tmn, mean transit time; IMR, index of microcirculatory resistance; IMRcorr, corrected IMR; LAD, left anterior descending artery; LCX, left circumflex artery; MVD, microvascular dysfunction; Pa (hyp), mean aortic pressure during hyperemia; Pd (hyp), mean distal coronary pressure during hyperemia; RCA, right coronary artery; Tmn, mean transit time; TIMI, thrombolysis in myocardial infarction; TMPG, thrombolysis in myocardial infarction myocardial perfusion grade.

**Table 3:** Clinical outcomes for 3-year MACE in study population.

Parameter	Total (*N* = 153)	High IMR (*N* = 52)	Low IMR (*N* = 101)	*P* value
3-years MACE, *n* (%)^*∗*^	17 (11.1%)	8 (15.4%)	9 (8.9%)	0.279
CV death	3 (2.0%)	3 (5.8%)	0 (0.0%)	0.038
Hospitalization for HF	1 (0.7%)	1 (1.9%)	0 (0.0%)	0.340
TLR	7 (4.6%)	1 (1.9%)	6 (5.9%)	0.424
Stent thrombosis	3 (2.0%)	1 (1.9%)	2 (2.0%)	1.000
Nonfatal MI	0 (0.0%)	0 (0.0%)	0 (0.0%)	1.000
Stroke	3 (2.0%)	2 (3.8%)	1 (1.0%)	0.267
Major bleeding	2 (1.3%)	2 (3.8%)	0 (0.0%)	0.114

CV, cardiovascular; HF, heart failure; IMR, index of microcirculatory resistance; MACE, major advanced cardiac events; MI, myocardial infarction; TLR, target lesion revascularization. ^*∗*^MACE was defined as composite of CV death, hospitalization because of HF, TLR, stent thrombosis, nonfatal MI, stroke, and major bleeding.

**Table 4:** Therapeutic strategies for STEMI in study population.

Parameter	Total (*N* = 160)	High IMR (*N* = 54)	Low IMR (*N* = 106)	*P* value
Mechanical strategies				
Stent diameter (mm)	3.0 [3.0–3.5]	3.0 [2.8–3.5]	3.0 [3.0–3.5]	0.248
Stent length (mm)	24.0 [18.0–30.0]	24.0 [22.0–33.0]	23.0 [18.0–28.0]	0.090
Pre balloon dilatation, n (%)	118 (73.8%)	37 (68.5%)	81 (76.4%)	0.377
Post balloon dilatation, n (%)	20 (12.5%)	9 (16.7%)	11 (10.4%)	0.376
Direct stenting, *n* (%)	42 (26.2%)	17 (31.5%)	25 (23.6%)	0.377
Thrombectomy, *n* (%)	75 (46.9%)	28 (51.9%)	47 (44.3%)	0.464
Thrombectomy + GP IIb/IIIa inhibitor, *n* (%)	31 (19.4%)	12 (22.2%)	19 (17.9%)	0.611

Pharmacological strategies				
Aspirin, *n* (%)	160 (100.0%)	54 (100.0%)	106 (100.0%)	1.000
Clopidogrel, *n* (%)	107 (66.9%)	43 (79.6%)	64 (60.4%)	0.023
Third-generation P2Y12 inhibitors, *n* (%)	53 (33.1%)	11 (20.4%)	42 (39.6%)	0.023
Morphine, *n* (%)	96 (60.0%)	28 (51.9%)	68 (64.2%)	0.183
GP IIb/IIIa inhibitor, *n* (%)	34 (21.2%)	15 (27.8%)	19 (17.9%)	0.216

GP, glycoprotein; IMR, index of microcirculatory resistance.

**Table 5:** Results of multiple linear regression analysis for clinical, echocardiographic, and angiographic factors correlated with IMR value.

Parameter	Univariable analysis			Multiple analysis		
	*β*	SE	*P* value	*β*	SE	*P* value
Age	0.35	0.14	0.017	0.23	0.16	0.538
Male	−3.53	5.13	0.493			
Killip class	13.65	3.10	<0.001	8.18	2.89	0.006
SHT^*∗*^	5.40	1.54	<0.001	3.88	1.33	0.004
LVEF	−0.67	0.23	0.005			
E/e'	0.78	0.58	0.183			
WMSI	17.12	5.49	0.002			
LV mass index	0.18	0.07	0.018			
CK-MB peak^*∗*^	2.64	1.55	0.093			
Troponin-I peak^*∗*^	3.91	1.44	0.008	2.52	1.22	0.042
NT-proBNP^*∗*^	5.08	1.43	<0.001			
Multivessel disease	11.47	3.44	0.001	9.09	2.93	0.003
Initial TIMI 0-1	5.92	3.62	0.105			
Final TIMI 3	−5.40	6.99	0.442			
Final TMPG 3	−8.10	3.76	0.034	−5.00	3.17	0.119
Distal embolization	14.17	4.44	0.002	8.38	3.88	0.034

*β*, Unstandardized coefficients; CK-MB, creatine kinase muscle/brain; LV, left ventricle; LVEF, left ventricular ejection fraction; NT-proBNP, *N*-terminal prohormone of brain natriuretic peptide; SE, standard error; SHT, symptom onset to hospital time; TIMI, thrombolysis in myocardial infarction; TMPG, thrombolysis in myocardial infarction myocardial perfusion grade; WMSI, wall motion score index. ^*∗*^Data were expressed as quartile (1^st^∼4^th^).

**Table 6:** Results of univariable and multiple linear regression analysis for therapeutic strategies correlated with IMR value.

Parameter	Univariable analysis			Multiple analysis			Multiple analysis^*∗*^		
	*β*	SE	*P* value	*β*	SE	*P* value	*β*	SE	*P* value
Mechanical strategies									
Stent diameter	−8.00	4.18	0.057	−9.39	3.94	0.018	−5.36	3.45	0.123
Stent length	0.31	0.15	0.046	0.23	0.14	0.118	−0.03	0.13	0.807
Pre-balloon dilatation	−3.64	3.24	0.264						
Post-balloon dilatation	10.44	4.25	0.015	11.40	4.22	0.008	8.30	3.53	0.020
Direct stenting	3.64	3.24	0.264						
Thrombectomy	2.60	2.87	0.366						

Pharmacological strategies									
Third-generation P2Y12 inhibitors	−7.24	2.99	0.017	−10.69	2.91	<0.001	−10.28	2.56	<0.001
Morphine	−4.08	2.92	0.164						
GP IIb/IIIa inhibitor	8.49	3.48	0.016	8.51	3.38	0.013	−0.98	3.50	0.756

*β*, unstandardized coefficients; GP, glycoprotein; SE, standard error. ^*∗*^Multiple regression analysis adjusted for age, sex, Killip class, symptom onset to hospital time, peak troponin-I level, multivessel disease, final thrombolysis in myocardial infarction myocardial perfusion grade, and presence of distal embolization.

## Data Availability

The data used to support the findings of this study are available from the corresponding author upon request.
